# Veno-occlusive disease nurse management: development of a dynamic monitoring tool by the GITMO nursing group

**DOI:** 10.3332/ecancer.2016.661

**Published:** 2016-08-08

**Authors:** Stefano Botti, Laura Orlando, Gianpaolo Gargiulo, Valentina De Cecco, Marina Banfi, Lorenzo Duranti, Emanuela Samarani, Maria Giovanna Netti, Marco Deiana, Vera Galuppini, Adriana Concetta Pignatelli, Rosanna Ceresoli, Alessio Vedovetto, Elena Rostagno, Marilena Bambaci, Cristina Dellaversana, Stefano Luminari, Francesca Bonifazi

**Affiliations:** 1 Haematology Unit, Arcispedale Santa Maria Nuova-IRCCS Reggio Emilia, Italy; 2 Division of Clinical Haemato-Oncology, Istituto Europeo di Oncologia, Milan, Italy; 3 Haematology and BMT Unit, AOU Federico II Napoli, Naples, Italy; 4 Paediatric Haemato-Oncology Unit, Policlinico S Matteo Pavia, Viale Camillo Golgi, 19, 27100 Pavia PV, Italy; 5 BMT Unit, Ospedale Maggiore IRCCS Milano, Milan, Italy; 6 Haematology and BMT Unit, Ospedale Silvestrini, Perugia, Italy; 7 BMT Unit, Spedali Civili di Brescia, Brescia, Italy; 8 SODc Paediatric Tumours and BMT Unit, Ospedale Pediatrico Meyer Firenze, Florence, Italy; 9 Paediatric Haematology/Oncology Department, IRCCS G Gaslini, Genova, Italy; 10 Quality Office, Istituto Regina Elena Rome Transplant Network, Rome, Italy; 11 Paediatric Haemato-Oncology and BMT Unit, Azienda Ospedaliera di Padova, Via Nicolò Giustiniani, 2, 35128 Padova PD, Italy; 12 Paediatric Haematology/Oncology and BMT Department, Azienda Ospedaliero, Universitaria S Orsola Malpighi, Bologna, Italy; 13 Paediatric Haemato-Oncology and BMT Unit , Ospedale Regina Margherita, Torino, Italy; 14 Haematology and BMT Unit, Azienda Ospedaliero, Universitaria S Orsola Malpighi, Bologna, Italy

**Keywords:** veno-occlusive disease, stem cell transplantation, nurse management, assessment, monitoring

## Abstract

Veno-occlusive disease (VOD) is a complication arising from the toxicity of conditioning regimens that have a significant impact on the survival of patients who undergo stem cell transplantation. There are several known risk factors for developing VOD and their assessment before the start of conditioning regimens could improve the quality of care. Equally important are early identification of signs and symptoms ascribable to VOD, rapid diagnosis, and timely adjustment of support therapy and treatment. Nurses have a fundamental role at the stages of assessment and monitoring for signs and symptoms; therefore, they should have documented skills and training. The literature defines nurses’ areas of competence in managing VOD, but in the actual clinical practice, this is not so clear. Moreover, there is an intrinsic difficulty in managing VOD due to its rapid and often dramatic evolution, together with a lack of care tools to guide nurses. Through a complex evidence-based process, the Gruppo Italiano per il Trapianto di Midollo Osseo (GITMO), cellule staminali emopoietiche e terapia cellulare nursing board has developed an operational flowchart and a dynamic monitoring tool applicable to haematopoietic stem cell transplantation patients, whether they develop this complication or not.

## Background

Veno-occlusive disease (VOD) or sinusoidal obstruction syndrome (SOS) is a potentially life-threatening complication affecting patients undergoing haematopoietic stem cell transplantation (HSCT) [1]. Conditioning regimens prior to HSCT favour endothelial cell activation, causing damage to sinusoids and liver cells [2]. Accumulation of cells and cell degradation products in the perisinusoidal space leads to an increased inflammation of the sinusoids, restricting their lumen through a thrombotic process until their complete obstruction. This rapidly results in slowing of intrahepatic blood flow that can go as far as inversion of the portal flow with compromise of liver function [1–3].

VOD has an average prevalence among patients undergoing HSCT of approximately 13.7%, [3] with a mortality of severe forms that reaches 80% of cases [4]. It is usually observed from 35 to 45 days after transplant, but it can also occur a long time afterwards. It is associated with an increase in HSCT-related costs [5–6] because of the need for intensive care.

There are several risk factors for the development of VOD that plays a role in increasing its incidence and impacting mortality [9–10], making it necessary to pay particular attention during patient assessment and monitoring activities [11–12].

Patient-related risk factors are as follows: liver disease, elevated transaminases, age, malignant pathologies, failure to achieve remission, history of hepatic irradiation, and genetic predisposition. Therapy-related risk factors are as follows: type of HSCT, intensity of conditioning, duration and type of drugs used and their hepatotoxicity, donor type and origin of stem cells, the presence of fever during conditioning, HSCT history, abdominal irradiation, and previous treatments with gemtuzumab [8–9].

VOD is diagnosed on the basis of clinical observation, using the Modified Seattle [15] and Baltimore criteria [16].

However, signs and symptoms vary and can be the expression of many other pathological conditions, which make differential diagnosis necessary [9,17,18]. There are many conditions that can mimic VOD, including acute hepatic graft-versus-host disease, fungal and viral infections, cholangitis, fatty liver disease, drug toxicity, and cholestasis. The differential diagnosis may present a challenge for the clinician, often with delayed treatment. Therefore, the early identification of signs of VOD is essential for setting up adequate treatment.

At the early stage, VOD is characterised by a hepatorenal syndrome, with rapid weight gain, ascites, hepatomegaly, jaundice, pain in the right upper quadrant, and thrombocytopenia [7–8] often followed by multiorgan failure (MOF) at the later stage.

VOD can be classified as follows on the basis of severity [18–19]:

- Mild VOD is defined as a pathology that meets the diagnostic criteria but does not require treatment for excess fluid or drugs for liver pain, the course of which is usually self-limiting;
- moderate VOD is a pathology with evidence of liver damage that requires treatment for excess fluid or drugs for liver pain, but which is fully resolved;
- severe VOD is defined as a pathology that causes death or is not resolved within 100 days following HSCT.

The above classification of VOD derives from retrospective studies and therefore is not useful from the prognostic point of view; some attempts, in this direction, were made by Chao [20] and Carreras [11] in 2014, but a strong consensus has yet to be found.

Progressive VOD may rapidly lead to MOF [15], with hepatomegaly and weight gain, progressive appearance of pulmonary and pleural infiltrates, diffuse oedema, cardiac insufficiency, renal insufficiency, ascites, bleeding, and neurological symptoms. Recovery from such a condition is very difficult [6].

Management strategies currently consist predominantly of a supporting therapy that takes into account the various organs involved and fluid and electrolyte balances remains one of the key elements in the phases of prevention and treatment of VOD [21].

Therapy with a combination of heparin and recombinant tissue plasminogen activator has shown responses in 30% of patients, but it is associated with an increased risk of haemorrhage and poor overall survival [17, 22–23]. Currently, the only drug approved by the European Medicines Agency (EMA) for the treatment of severe VOD is defibrotide [24]. Defibrotide has also been used in prevention with encouraging results [25, 26, 27, 28], although high-quality randomised, controlled studies are needed to define the ideal prophylactic regimen [29].

## Rationale

Every nurse working in a transplant programme (TP) should developed distinctive skills that exert a significant influence in terms of improving clinical outcomes, impacting on the quality and cost-effectiveness of treatments, and adding value to the nurse’s role itself in the transplant context [30].

Nurses are indeed involved during assessment and management of patients presenting with VOD, and a nurse trained to recognise signs and symptoms of VOD will certainly be able to contribute to early diagnosis of this complication [31–32]. The nurse role is fundamental during patient monitoring in order to report changes in weight, to assess jaundice, nutritional matters, and abdominal girth [32]. The nurse should be capable of correctly monitoring fluid and electrolyte balances, evaluating bleeding and assessing pain. Management of patients with VOD often requires fluid intake restriction, a personalised diet or fasting and patient mobilisation. It is also necessary to administer analgesics, blood products, electrolytes, and drugs, and to support the patient from the psychological point of view [17–18].

Joint Accreditation Committee-ISCT Europe (JACIE) [33] accreditation is a voluntary accreditation of excellence. JACIE quality standards require training and VOD management-specific skills only for physicians, although it is clear that the role of nurses is crucial for diagnosis and monitoring [18–34].

Different published studies [12, 17, 18, 34] describe the most important parameters to be assessed - suggesting the need of close monitoring, particularly in patients with severe VOD. However, to the best of our knowledge, this is the first report that provides a useful tool for clinical practice, differentiating VOD management on the basis of levels of risk and VOD evaluation. The aim of this work is to describe the development of a new tool ‘VOD early identification and management’ by the Gruppo Italiano Trapianto di Midollo Osseo Nursing Group (GITMO NG).

## Materials and Methods

The tool was created using the following three different routes:

- Creation of a nurse expert panel (NEP)
- Critical analysis of the literature and standard operating procedures (SOPs)
- Analysis of daily clinical practice in TPs (multicentre survey)

During the 2014 GITMO congress, the nurse board formed an expert panel (NEP), made up of professionals with expertise in stem cell transplantation from various Italian TPs. The NEP consisted of 10 nurses working in paediatric TPs and eight nurses working in adult TPs, including three specialist paediatric nurses, two research nurses, six head nurses, and one quality manager nurse. The mean age of the group was 42 years (28–57); the average length of service in TP of 14.8 years (6–28).

A literature search was conducted on PubMed, Embase, Cinhal, Cochrane Library, National Guideline Clearinghouse, the National Institutes of Health, and afterwards, the group performed a critical review of published studies. In addition, the group reviewed SOPs in the use in TPs and performed an audit of clinical competencies and activities to assess and monitor HSCT patients.

At the same time, a survey questionnaire was developed and shared among all Italian TPs, with the aim of taking a snapshot of the reality of clinical practice on management of VOD in patients undergoing allogeneic HSCT. The survey consisted of 43 questions, with single or multiple responses, addressing organisational and structural topics, monitoring of patients undergoing HSCT, nursing skills and knowledge related to VOD management (assessment, diagnosis, prevention, treatment, care). The survey was shared with all the transplant centres in the GITMO network on a web platform, and data were analysed by frequency using SPSS 20.

The results of the literature and SOPs reviews, the audit and the survey were shared and discussed within the NEP in some structured meetings.

The NEP evaluated the results and identified the key points for the development of the tool to assess and monitor patients throughout their transplant journey. Then, the NEP produced a pathway flow chart.

## Results

### Results of the multicentre survey

‘The nurse in HSCT: monitoring hepatic complications and management of sinusoidal obstruction syndrome (SOS) and veno-occlusive disease (VOD)’.

The survey conducted within the GITMO network was addressed to centres that carry out allogeneic HSCT. The main results revealed a somewhat mixed approach to monitoring across the various TPs; the Seattle and Baltimore diagnostic criteria are not often used by nurses, there appears to be a substantial lack of specific tools and patient monitoring is done through standard procedures for the general assessment of patients after transplantation. TPs characteristics are given in Table 1.

### Standard monitoring

**Figure 1.**
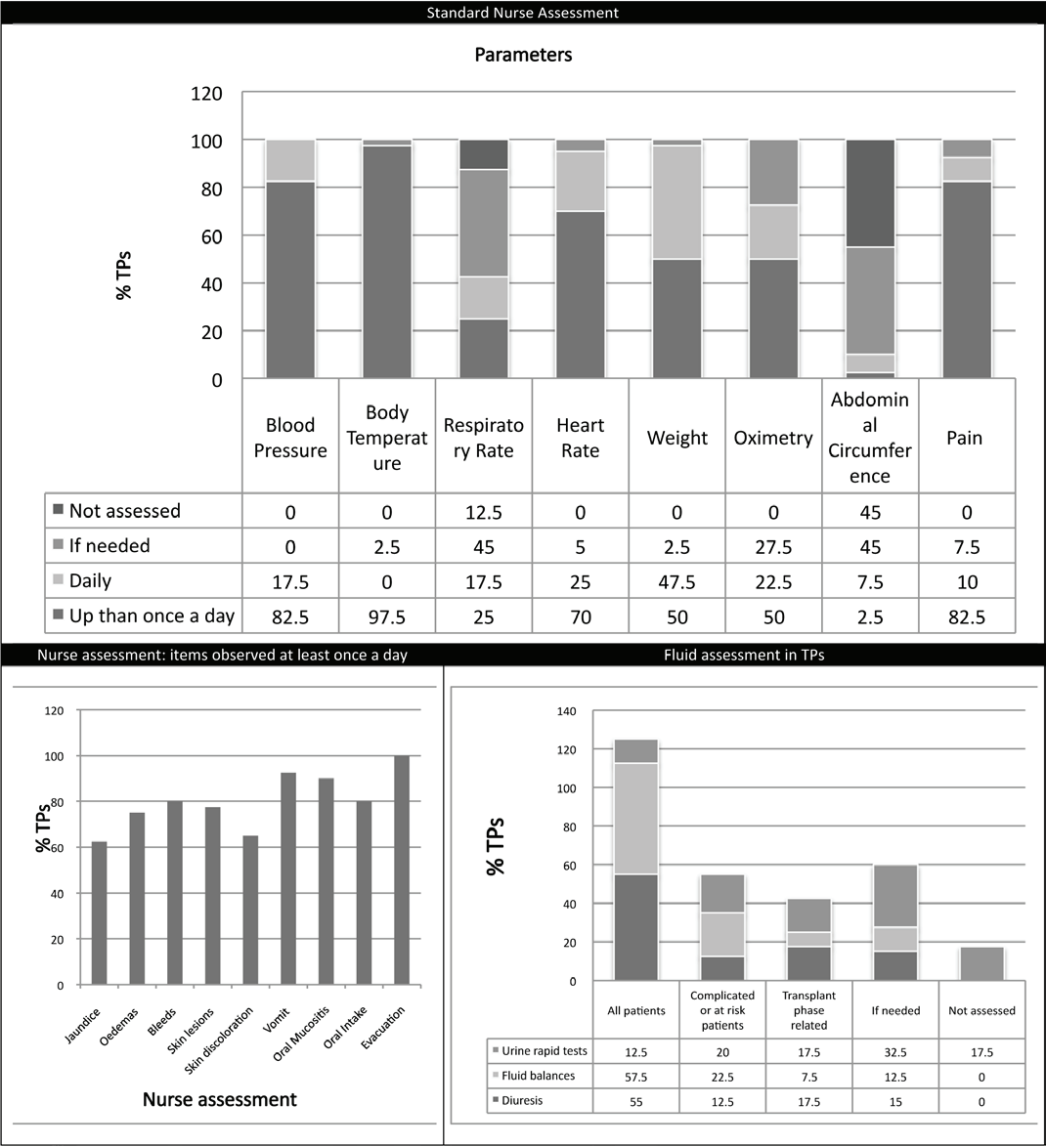
Standard nurse assessment.

The vast majority of TPs (95%) have monitoring protocols or specific checklists (82.5%). The parameters that are assessed at least daily (1 to 3 times per day) in all centres are classically: blood pressure, heart rate, temperature, weight, and pain. There are wide differences in the approach regarding the assessment of respiratory rate, oximetry, and diuresis. Figure 1 shows the main results related to standard monitoring practice in TPs. Only 40% of centres report written documentation of alert values derived from monitoring data. For the purposes of this paper, we are able to observe that only 10% of centres put daily measurement of abdominal girth as a standard monitoring parameter and that no centres take into account any other type of anthropometrical measurement (e.g., deltoid).

All the centres check fluid and electrolyte balances, but with widely differing policies (Figure 1). Ninety-five per cent of transplant programmes do not have nurses trained in abdominal palpation of patients; in only 5% of TPs abdominal palpation is also carried out by nurses.

**Figure 2.**
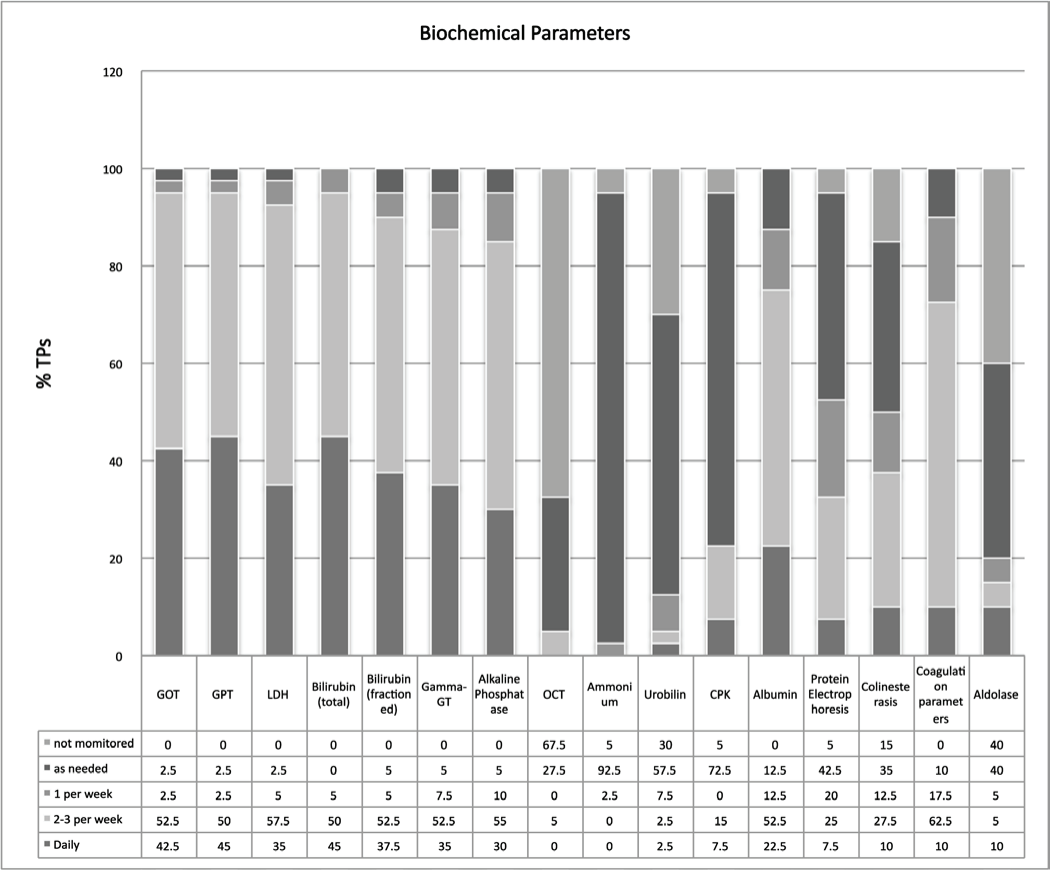
Standard monitoring of biochemical parameters.

More than 90% of TPs carry out laboratory tests more than 2–3 times a week. In this context, we also noted that parameters useful in the diagnosis of VOD are not recorded in a structured and systematic manner: aldolase is monitored on a routine basis only in 20% of TPs, and ornithine transcarbamylase only in 5%. Figure 2 shows detailed data on the standard monitoring of biochemical parameters.

### Nurse training

In 40% of transplant programmes, nurses had the opportunity to receive training in hepatic complications of HSCT within the last two years, but the participation rate of nurses in training events was lower than 60% in 68.8% of TPs.

### VOD patients management

It was found that pre-HSCT risk assessment for hepatic complications is not carried out in 15.4% of TPs. The overall risk assessment is usually carried out by physicians without the presence of a nurse (85%) and in 33.3% of TPs nurses are not informed of the results of the pre-HSCT risk assessment.

In 82.5% of TPs, SOPs for the diagnosis of VOD are present and in 90% of centres the modified Seattle and Baltimore criteria are used by healthcare professionals. However, in 82.5% of TPs, the Seattle and Baltimore diagnostic criteria are not used by nurses. There are SOPs for the prevention and the treatment of VOD in 55% and 82.5% of TPs, respectively. Nurses are familiar with the differential diagnostic pathway (72.5% of TPs), the strategies adopted to prevent VOD (65%), and therapeutic strategies (80%).

Despite this, availability of SOPs for care (42.5%) and monitoring (52.5%) of patients with VOD is scattered in Italian TPs.

The most widely used techniques for differential diagnosis remain hepatic vessel Doppler (95%), hepatic tomography (30%) and levels of plasminogen activator inhibitor-1 (30%). Only 7.5% of TPs use transjugular biopsy to diagnose VOD.

For prevention strategies, our results showed a certain amount of variation, with 35% of TPs not undertaking pharmacological prevention, and the remaining centres using various strategies, among which the most representative are ursodeoxycholic acid, supportive therapy, and defibrotide.

Supportive therapy and defibrotide seem to be the therapeutic strategies universally used.

### Results from the NEP

The NEP, through the literature analysis and sharing of procedures and experiences, identified three key areas for the assessment and monitoring of patients, corresponding to the clinical phases of the transplant journey: pre-conditioning period, during conditioning and post-conditioning. To allow for applicability in the various treatment settings, essential parameters were identified and agreed by the group. A full account of nursing activities in the various degrees of VOD is described by Eisenberg *et al* [18].

### Pre-conditioning (or assessment phase)

Generally, before admission, an assessment process is put in place through a multidisciplinary approach; nurses should be involved and should be informed about results of risk assessment. The group agreed that all patients undergoing HSCT should be considered at risk of VOD, whereby the NEP decided to identify two risk levels: ‘standard risk’ to be assigned to all patients without major risk factors for developing severe VOD, such as the presence of active and previous pathologies of the liver (fibrosis, cirrhosis, fatty liver disease, previous hepatitis), high transaminase levels also in the absence of other clinical signs of level disease, allogeneic HSCT, myeloablative conditioning, the use of drugs such as busulfan, the use of total body irradiation (TBI), alloreactivity, previous hepatotoxic treatments (gemtuzumab) [11–17]. The expert panel defined at ‘increased’ risk patients in whom at least one of the factors listed previously is present or a combination of other risk factors such as age and sex (elderly women are at greater risk), type and status of disease, genetic susceptibility, development of fever during conditioning, hepatotoxic treatments (vancomycin, acyclovir). At this phase, it is important that nurses perform anamnesis and assessment by collecting information and checking parameters in the areas indicated in Table 2 (part A). Results of the nursing assessment process should be shared within the team, recorded in patient records and reviewed, if necessary, before beginning conditioning. Implementation of alert systems may be also useful in this setting.

### During conditioning

For this period, at this stage, the expert panel defined two monitoring protocols, as shown in Table 2 (part A), of differing intensity according to the level of risk established at the previous phase.

### After conditioning

During this period, nurses should carry out systematic assessment using Seattle (modified) and Baltimore criteria. If no change from baseline values is detected, patient monitoring should be continued routinely.

If on the basis of Seattle and Baltimore criteria a ‘Suspected VOD’ is define, physicians should be alerted to begin differential diagnosis procedures as soon as possible.

It is also necessary to make arrangements for the intensification of the monitoring process as indicated in Table 2 (part B), until the diagnosis is confirmed or ruled out, paying particular attention to the rate of progression of disease and multiple organ involvement.

If differential diagnosis confirms the presence of VOD, patient monitoring should be intensified (high-intensity monitoring protocol) until continuous monitoring of certain parameters is introduced, as indicated in Table 2 (part C).

Activities for intensive support in relation to any multiple organ components should be put in place and preparations made for a quick transfer of the patient to intensive care (ICU). If VOD is not confirmed, care will continue in accordance with the clinical standards for whatever is present. The expert panel agreed that if the differential diagnosis is complex and/or it is not possible to have a diagnostic confirmation of VOD, faced with a rapid worsening of the patient’s signs and symptoms it is necessary to consider the patient critical and go ahead with high intensity monitoring and possible transfer of the patient to the ICU.

## Conclusions

Patient assessment and monitoring represent the mainstream of effective nursing support. It is fundamental to support these activities with specific tools, suited to the setting of care, in order to avoid situations where data and information are not appropriately recorded therefore producing a negative impact on the care process. In HSCT, the patient’s clinical conditions are extremely variable and consequently require tools capable of capturing all the possible changes that can have an impact on the patient’s health.

The early diagnosis and management of VOD is complex. It is essential that standardised, evidence-based practice procedures, which are ultimately based on practical needs and on clinical experience, are not perceived only as theoretical models but instead as widely accepted daily practices.

**Figure 3.**
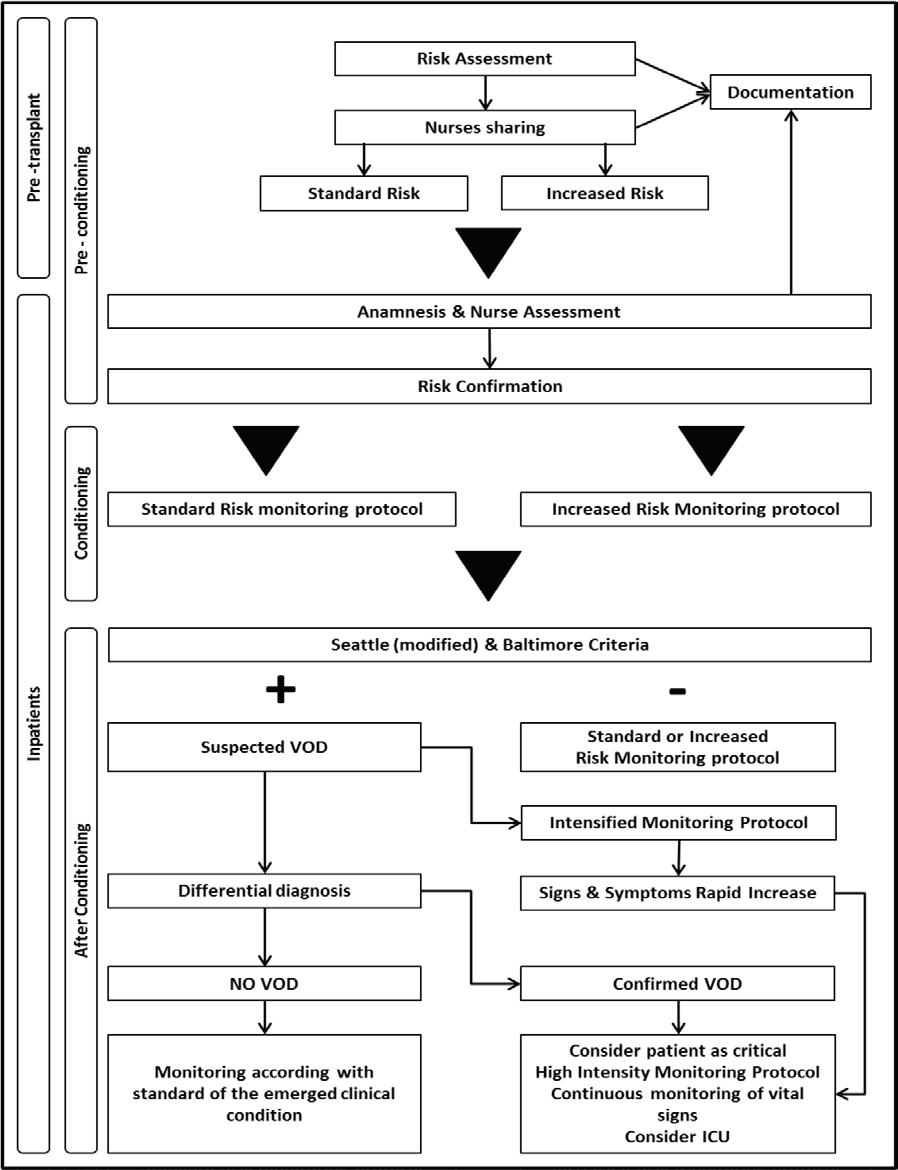
Pathway flow chart.

Eisenberg’s suggestions [18] for appropriate nursing interventions to support the various organs and tissues and the psychological state of patients with VOD, were very helpful in this respect. Despite this, an unequivocal guidance for VOD-related nursing problems is not available. This work is aimed at defining dynamic and practice guidelines taking into account the evolution of the patient’s conditions and the need of a practical approach in the transplant setting. In conclusion, the NEP on behalf of the GITMO NG is proposing a practical and dynamic flow chart that could be useful for the assessment and monitoring of the patient clinical course (Figure 3). This tool is easily manageable and could be immediately adopted in daily practice. In view of the innovative nature of this tool, a prospective clinical evaluation is warranted.

## Figures and Tables

**Table 1.** Characteristics of transplant centres.

	Responses (%)
Number of respondents	40/80 (50.0)
**Patient group**	
Adults Paediatrics Both (adults and paediatrics)	24 (60.0) 11 (27.5) 5 (12.5)
**Centre organisation**	
SCT unit SCT unit within Haematology unit	17 (42.5) 23 (57.5)
**Transplant type performed**	
Autologous – Sibling Autologous – Allogeneic – MUD All transplant therapies	2 (5.0) 5 (12.5 33 (82.5)

SCT: stem cell transplant; MUD: matched unrelated donor

**Table 2.** Monitoring protocol.

**Part A. Case history/Pre-conditioning nursing assessment**		
Take vital signs: arterial pressure, heart rate, respiratory rate, body temperature, oxygen saturation Measure weight (define 5% threshold value) Abdominal assessment (visual and by palpation), in particular of the RUQ: record spontaneous and induced pain (Blumberg), record abdominal circumference, abdominal volume, the presence of collateral circles and/or spiders, tractability, percussion (obtuseness), the assessment of hepatic RIMA and dimensions of the liver, assessment of hepatic consistency Assessment of skin: erythema, lesions, haemorrhages, dyschromia (jaundice) Assessment of sclera: microhaemorrhages, jaundice Consider blood test values: total and fractionated bilirubin, transaminases, LDH, electrolytes (Na, K) Details of patient’s clinical history, personal habits (diet, smoking, alcohol) and social background		
Assignment of standard or increased risk level Alert for the confirmation of risk level on admission		
**Standard risk**		**Increased risk**
**At least once a day ** Weight Abdominal circumference Abdominal pain and RUQ pain Objective examination of skin, sclera, abdomen (palpation) Fluid balance **At least twice a week and whenever significant variations in clinical signs are observed** Measurement of bilirubin, transaminases, sodium, potassium, coagulation parameters		**At least twice a day** Weight State of awareness Abdominal circumference Abdominal pain and RUQ pain Objective examination of skin, sclera, abdomen (palpation) Fluid balance **Between three times a week to every day whenever significant variations in clinical signs are observed** Measurement of bilirubin, transaminases, sodium, potassium, coagulation parameters
Report even small changes in clinical condition in a timely fashion Educate patient and CGs in timely communication		Report even small changes in clinical condition in a timely fashion Educate patient and CGs in timely communication
**Part B. Patients who meet the modified Seattle and Baltimore criteria: Intensified Monitoring Protocol**		
**At least twice a day** State of awareness Abdominal circumference Full objective examination of abdomen, sclera, skin, and mucosae	**At least three times a day** Weight Pain in RUQ Monitor for appearance of signs of haemorrhage (skin, sclera, mucosae	**At least four times a day** Fluid and electrolyte balance Oxygen saturation Vital signs: arterial pressure, heart and respiratory rate, body temperature
Foresee the need for continuous monitoring of parameters Intensify monitoring of blood tests: fractionated bilirubin, transaminases, coagulation parameters, sodium, potassium, and other tests prescribed by the doctor Provide psychological support for patient and family members or CGs		
**Part C. Patients with a diagnosis of VOD: High-intensity monitoring protocol**		
**At least twice a day** Abdominal circumference Full objective examination of abdomen, sclera, skin and mucosae	**At least three times a day** Weight Pain in RUQ Monitor for the appearance of signs of haemorrhage (skin, sclera, mucosae)	**At least four times a day** Fluid and electrolyte balance
**Consider patient as critical**		
Continuous monitoring of vital signs using monitors: arterial pressure, heart rate, respiratory rate, body temperature, oxygen saturation. Ventilatory support if necessary: oxygen therapy or non-invasive ventilation Ensure drastic reduction in fluid intake Ensure appropriate number of vascular access points Frequently monitor diuresis, possible use of bladder catheter with urometer Evaluate performance status and state of awareness Monitor for MOF: cardiac, respiratory, and renal function Provide psychological support for patient and family members or CGs Make arrangements for rapid transfer to CU		

RUQ: right upper quadrant; LDH: lactic dehydrogenase; CGs: care givers; MOF: multiple organ failure; ICU: intensive care unit.
